# Expression of Foreign Genes Demonstrates the Effectiveness of Pollen-Mediated Transformation in *Zea mays*

**DOI:** 10.3389/fpls.2017.00383

**Published:** 2017-03-21

**Authors:** Liyan Yang, Guimei Cui, Yixue Wang, Yaoshan Hao, Jianzhong Du, Hongmei Zhang, Changbiao Wang, Huanhuan Zhang, Shu-Biao Wu, Yi Sun

**Affiliations:** ^1^Biotechnology Research Center, Shanxi Academy of Agricultural SciencesTaiyuan, China; ^2^College of Life Science, Shanxi Normal UniversityLinfen, China; ^3^Maize Research Institute, Shanxi Academy of Agricultural SciencesTaiyuan, China; ^4^School of Environmental and Rural Science, University of New England, ArmidaleNSW, Australia; ^5^Key Laboratory of Crop Gene Resources and Germplasm Enhancement on Loess Plateau, Ministry of AgricultureTaiyuan, China

**Keywords:** pollen-mediated transformation, sonication, eGfp, Cry1Ac, *Zea mays*

## Abstract

Plant genetic transformation has arguably been the core of plant improvement in recent decades. Efforts have been made to develop in planta transformation systems due to the limitations present in the tissue-culture-based methods. Herein, we report an improved in planta transformation system, and provide the evidence of reporter gene expression in pollen tube, embryos and stable transgenicity of the plants following pollen-mediated plant transformation with optimized sonication treatment of pollen. The results showed that the aeration at 4°C treatment of pollen grains in sucrose prior to sonication significantly improved the pollen viability leading to improved kernel set and transformation efficiency. Scanning electron microscopy observation revealed that the removal of operculum covering pollen pore by ultrasonication might be one of the reasons for the pollen grains to become competent for transformation. Evidences have shown that the *eGfp* gene was expressed in the pollen tube and embryos, and the *Cry1Ac* gene was detected in the subsequent T_1_ and T_2_ progenies, suggesting the successful transfer of the foreign genes to the recipient plants. The Southern blot analysis of *Cry1Ac* gene in T_2_ progenies and PCR-identified *Apr* gene segregation in T_2_ seedlings confirmed the stable inheritance of the transgene. The outcome illustrated that the pollen-mediated genetic transformation system can be widely applied in the plant improvement programs with apparent advantages over tissue-culture-based transformation methods.

## Introduction

Genetic transformation has become an important tool in the improvement of plants. Numerous genetic transformation methods have been developed in the past three decades for a wide range of plant species. Generally, they have been classified into indirect or direct transformation (Rao et al., 2009). Biological methods using bacteria or viruses are referred to as indirect, while direct methods are basically in physical ways, that is, by the penetration of the cell wall (Torney et al., 2007; Rivera et al., 2012; Ziemienowicz et al., 2012). At present, two transformation methods are most commonly used in plants, i.e., *Agrobacterium*-mediated transformation and particle bombardment. Although widely used in the research community, these methods have a common disadvantage, i.e., the requirement of a long and laborious plant tissue culture process, which is highly genotype-dependent and costly (Birch, 1997; Ioannidis et al., 2016). As some plant species or varieties are difficult to regenerate through tissue culture, the applications of these methods have been restricted to some extent. Furthermore, the occurrence of somatic variation and low survival rate of some regenerated plantlets during transplanting process make the already low transformation efficiency further compromised. Despite of some reports on transformation approaches independent of tissue culture, these methods have not been applied efficiently in practice due to the complexity to operate and low efficiency. Therefore, transformation methods without tissue culture procedure and simple to operate are needed.

The interest in developing non-tissue culture based or *in planta* transformation methods has been growing (Shou et al., 2002). The production of transgenic plants in such way could in general be relatively simple, inexpensive and efficient. Although being independent of tissue culture and having been used for many years, floral dipping method is confined to only a few species that also restricted its wider application in many other plant species (Clough and Bent, 1998). It has been considered, therefore, that the reproductive system of plants is a potential pathway for the introduction of foreign genes (Luo and Wu, 1989).

Attempts have been made to develop non-tissue culture based transformation in plants in the last three decades (Ohta, 1986; Booy et al., 1989; Li et al., 2004). Pollen tube pathway-mediated genetic transfer was reported in genetic transformation in cotton (Zhou et al., 1983) and rice (Luo and Wu, 1989). This method was once regarded advantageous due to its independency of tissue culture and plant regeneration, no requirement on well-equipped labs, and its simplicity to operate. However, the transformation efficiency of this method has been proven low and thus requires massive transformant selection efforts among a large number of progenies, and the method was not reproducible at least in some cases (Shou et al., 2002). Furthermore, Booy et al. (1989) demonstrated that the digestion of plasmid DNA by nuclease was a major obstacle for using pollen grains as vector for genetic transformation.

It has been suggested that the ultrasonication treatment of pollen grains denatures the nuclease while not completely inhibiting their viability and germination, and a genetic transformation approach using plant pollen as a vector was reported (Wang et al., 2001). In the method, pollen grains are targeted as a vector to carry foreign genes with the assistance of sonication treatments in a sucrose solution. Genes of interest were then incorporated to the progenies by pollination process with the treated pollen and subsequent selection of genetically transformed seedlings. Pollen-mediated transformation is a quick and easy alternative method to generate transgenic plants, bypassing the requisite for tissue culture. It has a potential to produce genetically modified plants within a short period, leading to inclusion of the method in conventional crop breeding programs. The method has also been proven applicable in sorghum (Wang et al., 2007) and *Brassica* (Wang et al., 2008). Nevertheless, a major shortcoming of the then reported method showed low kernel setting rate after pollination, because most pollen grains lose their viability after ultrasonication and fail to complete the fertilization process. Further, evidences of transformation in the following procedures, for example, during pollen germination, and embryo development were lacking to support the success of transformation was due to the transformation of pollen grains. Therefore, improved kernel setting rate and further evidences to demonstrate the transformation of pollen grains as vectors have become desirable for wider application of such method. We hypothesize that low temperature and aeration may be beneficial for maintaining pollen viability during sonication process as pollen is active in metabolisms which requires large amount of oxygen and temperature may rise. Therefore, we tested the effects of aeration and low temperature treatment to the sucrose solution prior to transformation of pollen grains. Further, we also hypothesize that foreign gene (s) transformed to generative cell/sperms will be incorporated to zygote through fertilization of egg by the sperm with transgene and then passed on to the progeny. Herein, we report an optimized pollen-mediated plant transformation method that demonstrated effectiveness of the transformation of foreign genes by their expression at various stages.

## Materials and Methods

### Plant Materials

Maize inbred Zheng58 and Dika527 obtained from Qiangsheng Seed Ltd. Shanxi, China, were used throughout the study. The plants were grown at an isolated experimental plot in the Experimental Research Station of Shanxi Academy of Agricultural Sciences, Taiyuan, China (N37°46′, E112°34′), and tassels of recipient maize plants were removed before anthesis to keep a pollen-free condition. Pollen donor maize plants were grown in another experimental plot which was more than 500 m apart from the recipient. The seeds were sown in early May and corn ears were harvested in early October each year with sufficient irrigation when needed. The field management was carried out according to the guideline provided for the breed.

### Transformation Vectors

**FIGURE 1:**
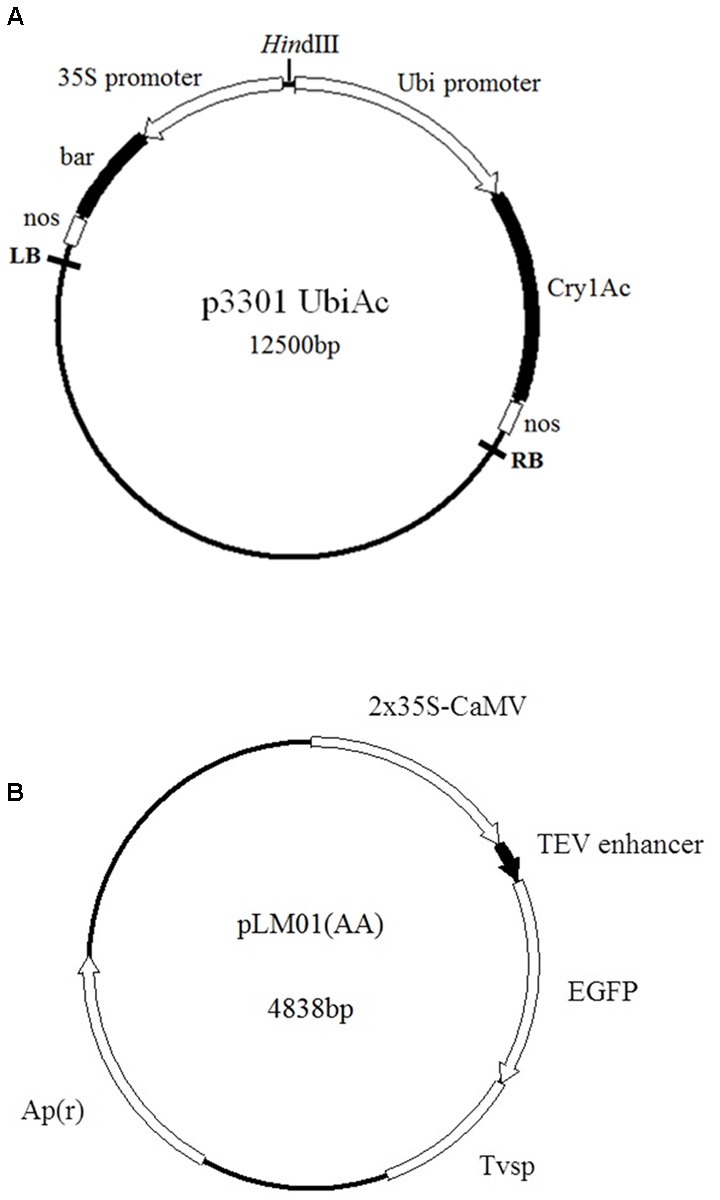
**Maps of plasmids employed in the transformation. (A)** p3301UbiAc: the *bar* gene is controlled by a cauliflower mosaic virus (CaMV) 35S promoter (35S) and a nos terminator; the *Cry1Ac* gene is controlled by a ubiquitin promoter (Ubi, Z*ea mays*, Accession No. S94466) and a nos terminator; **(B)** pLM01: the *eGfp* gene is controlled by two CaMV 35S promoters (P35S), a tobacco etch virus 5′- untranslated region (TEV) enhancer, and a soybean vegetative storage protein terminator (Tvsp) followed by an ampicillin (*Apr*) resistant marker gene for the selection in *Escherichia coli* culture.

Two constructs, plasmid p3301UbiAc harboring a bar gene as a selector and a gene *Cry1Ac* kindly provided by Lian et al. (2008), and plasmid pLM01 harboring an ampicillin resistant gene (*Apr*) as a selector and an *eGfp* gene gifted by Moeller et al. (2009) (**Figure 1**), were used in the present study. Plasmid DNA was extracted with alkaline lysis according to Sambrook et al. (1989).

### Pollen Grain Treatment

#### The Pollen Collection

In order to be able to harvest desirable quality of fresh pollen, old anthers were removed and the tassels were bagged in the afternoon the day prior to the collection. On the collection day, fresh pollen grains were collected into a paper bag between 10:00–13:00 by shaking the tassels. The collection was sieved to remove anthers, and the pollen grains were used immediately for the downstream treatments or preserved in Petri dishes placed in a hydro-prevent chamber at 4°C until required.

#### Aeration and Temperature Treatment Optimize for Pollen Grains

**FIGURE 2:**
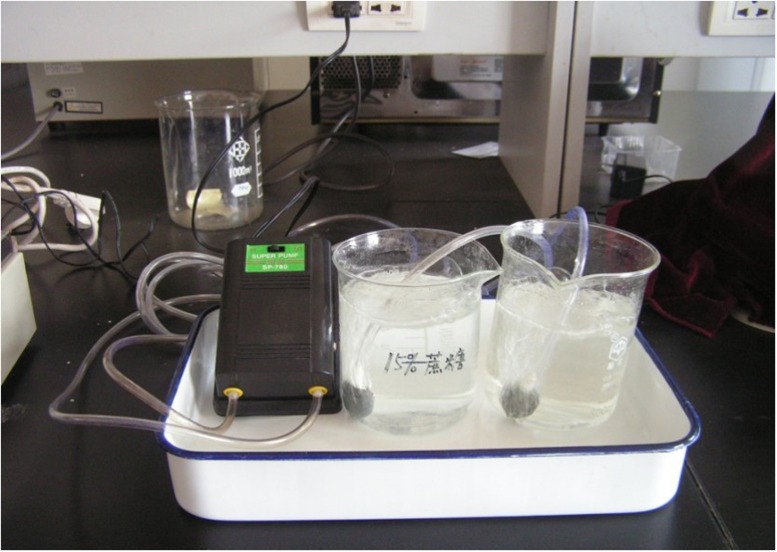
**Aeration treatment of sucrose solution for suspending maize pollen.** Sieved pollen was suspended in 15% sterilized sucrose solution in a beaker and the suspension was aerated by an air pump for 20 min prior to ultrasonication treatment.

A 2 × 2 factorial design was performed to test the effect of aeration and temperature treatments on pollen viability and germination capability. The factors were: aeration treatment, 0 min or 20 min and temperature, 4°C or 25°C. Here, the group with 0 min aeration and temperature at 25°C was regarded as an experimental control. A total of 0.3 g sieved pollen was suspended in 20 ml of 15% sterilized sucrose solution in a beaker. The suspension was aerated by an air pump (**Figure 2**) for desired time at desired temperature as indicated previously as the treatments. Approximately 500 μl of the pollen suspension or equivalent to 3.0 × 10^4^ pollen grains (247 × 10^-9^ g / maize pollen grain; Miller, 1982) was allocated to a small Petri dish (3 cm in diameter) to examine the germination rates by incubation for 2 h at 25°C. Pollen intactness and pollen tube growth were observed with an optical microscope (IX51, Olympus, Osaka, Japan). Eight replications were taken with 60 pollen grains for each image.

#### Sonication Treatment

The best combination of aeration time and temperature showing optimal pollen intactness, germination rate, and pollen tube growth was applied to pollen suspension before a sonication pre-treatment, then the pollen grains were treated with an ultrasonic homogenizer (Ningbo Scientz Biotechnology Co. Ltd., China, JY92-llN; Frequency: 20–25 Hz) by submerging the probe of the homogenizer into the pollen suspension. The power of the ultrasonic homogenizer was set to 150 W and the sonication lasted for 10 s each time with an interval of 10 s for a total of six times.

Ten microgram of plasmid DNA (approximately 1: 30,000 W/W of DNA: pollen) was added to the treated pollen suspension and then sonicated again as described above. All the sonication procedures were performed on ice to protect DNA and pollen contents from being degraded or damaged. Untreated pollen mixed with plasmid DNA was used as control.

### Pollination

The treated pollen suspension was incubated at room temperature for 10 min following the ultrasonication to let the pollen grains to sediment and the supernatant was then carefully discarded. The pollen mass obtained was gently brushed onto the silks of recipients. The pollinated ears were then bagged to protect stray pollen contamination. The mature ears were harvested and the seeds were collected and stored at room temperature until they were sown in the following year. Approximately one thousand maize ears were pollinated and harvested for each treatment, and seed setting were recorded for each ear.

### Transformant Screening

In contrast to the transformation of somatic cells, we define the primary transgenic kernels as T_1_ kernels and the seedlings from the kernels are T_1_ plants. Harvested T_1_ kernels were sown in the field as described. At 5–6 leaf stage, fresh leaves from T_1_ seedlings were collected (for plants pollinated with pLM01 DNA) or sprayed with herbicide Basta (50 μl L^-1^) to select survived plants (for plants pollinated with plasmid p3301UbiAc DNA ), and total DNA was extracted with CTAB method for subsequent molecular analysis (Doyle, 1991).

### Scanning Electron Microscopy (SEM)

Pollen grains from sonication and control groups were fixed in 2% glutaraldehyde solution (0.2M, pH7.1 phosphate buffer) and kept at 4°C until further processing. Pollen grains were dehydrated in gradient alcohol solutions at the concentrations of 30, 50, 70, 90, and 100%, each for 10 min successively. The dehydrated samples were then dried at the ambient temperature overnight, and coated with gold using a JEOL JFC-1600 AUTO COATER. The morphology of the pollen grains was viewed and images taken under a scanning electron microscope (JEOL JSM-7500, Field Emission Scanning Electron Microscope, JEOL, Ltd Tokyo Japan).

### Fluorescence Microscopy and Imaging

Pollen, pollen tube, and embryo were observed and photographed under bright field with white light illumination using an Olympus Microscope (U-LH100HG, Olympus, Osaka, Japan). *EGfp* expression was observed under fluorescence microscope at 0.5 h interval with blue light (495 nm) as the exciting light. A drop of pollen suspension from various treatment groups was incubated at 25°C on the glass slide kept in a covered Petri dish containing wet filter paper. Pollen tubes were observed 2.5–3 h after sonication treatment. Embryos were excised with a scalpel harvested at 15 days after pollination and then observed and photographed under bright field and fluorescence microscope as described above.

### Polymerase Chain Reaction

Polymerase chain reaction (PCR) was performed to detect the presence of *Cry1Ac* or *Apr* gene fragment with primer pairs (Forward: 5′- CTG ACC GTG ACC GTG CTG -3′ Reverse 5′- TGG the TGC CGT AGG CGA ACT -3′, which was ∼500bp, or Forward: 5′- ATGAGTATTCAACATTTCCG -3′ Reverse 5′-TTTGGTATGGCTTCATTCAG -3′, which was ∼500 bp. The primers were synthesized by Sangon Biotechnology Company, Shanghai, China. PCR was performed in a volume of 25 μl consisting of 2.5 μl 10× reaction buffer, 200 mM dNTPs, 15 mM MgCl2, 100 ng of DNA, 0.5 μM of each primer, and 0.5 U of Taq DNA polymerase (TaKaRa), using a PTC-200 Thermal Cycler (MJ Research, Watertown, MA, USA). The PCR cycles were: initial denaturation at 94°C for 5 min, then 30 cycles of 94°C for 30 s, 56°C for 45 s, 72°C for 30 s, and a final extension at 72°C for 10 min. The amplified products were electrophoresed on 1% agarose gel, visualized and photographed under ultraviolet light following ethidium bromide staining.

### Southern Blot

Southern hybridization was performed with a *Cry1Ac* specific probe. Plasmid P3301UbiAc DNA was used as PCR template with the primers 5′- CTG ACC GTG ACC GTG CTG -3′(Forward) and 5′-TGG TGC CGT AGG CGA ACT -3′(Reverse), and the probe was labeled with digoxigenin which was ∼500 bp. Approximately 15 μg genomic DNA of the PCR-positive T_2_ and control plants were purified as described and digested overnight, with *Hind* III endonucleases (*Hind* III cuts once in the plasmid). And the digested DNA was then separated on a 1% agarose gel followed by transferring the fragments to a nylon membrane. The membrane was subjected to hybridization over night with digoxigenin labeled probe that was then detected by the digoxigenin luminescence detection procedure according to the instruction of Roche DIG High Prime DNA Labeling and Detection Starter Kit II (Roche Applied Science, Penzberg, Upper Bavaria, Germany).

### Statistical Analysis

Pollen viability and kernel set data were analyzed using the statistical package IBM^®^ SPSS^®^ Statistics package, version 19 (IBM Corporation). The main effects of aeration and temperature, and their interactions were examined by analysis of variance using the general linear model. The data of individual treatments were subjected to one-way ANOVA analysis to determine the optimal treatment conditions.

## Results

### Effects of Aeration and Temperature on Pollen Viability and Kernel Set

**FIGURE 3:**
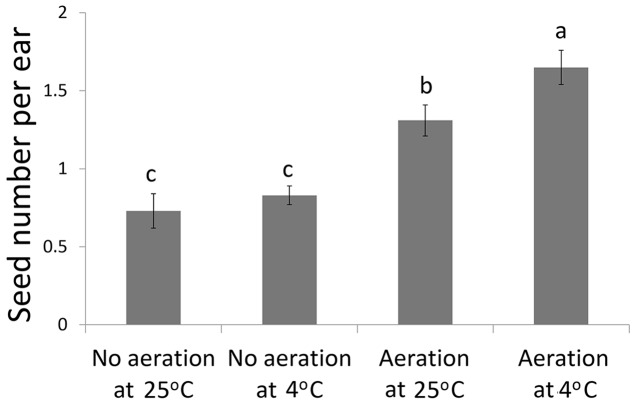
**Kernel set per ear on recipient plants following pollination of the sonication treated pollen with or without aeration at 4°C or 25°C prior to transformation of the foreign genes**.

Aeration of the sucrose solution significantly reduced the broken rate of pollen grains (*P* < 0.001), improved pollen germination rate (*P* < 0.01) and increased the pollen tube length (*P* < 0.05) (**Table 1**). Similar effects were also observed following lower temperature (4°C) treatment with significantly reduced pollen broken rate (*P* < 0.05), improved germination rate (*P* < 0.001), and longer pollen tube (*P* < 0.01). Overall, the combination of aeration for 20 min at 4°C produced best pollen performance following sonication treatment, i.e., lowest broken rate (24.5%), highest germination rate (11.91%), and longest pollen tube (1528 μm). Furthermore, aeration treatment of pollen prior to sonication for 20 min at 4°C significantly increased kernel set of the recipient ears pollinated with the treated pollen (**Figure 3**). Therefore, this optimal condition was used for the subsequent transformation procedure.

**Table 1:** Effects of aeration and temperature prior to sonication on pollen germination and pollen tube growth of maize.

Treatment							
Aeration	Temperature	Broken rate (%)^∗^	*SE*	Germination rate (%)^∗^	*SE*	Length of pollen tube (μm)^∗^	*SE*
No	25°C	80.1^a^	8.2	3.74 ^c^	1.22	822^c^	100
No	4°C	61.0^b^	6.8	8.19 ^b^	1.36	1378^ab^	137
Yes	25°C	32.5^c^	4.2	6.03 ^b^	1.23	1222^b^	113
Yes	4°C	24.5 ^c^	3.4	11.9 ^a^	2.39	1528^a^	150
**One-way ANOVA (P)**		*P* < 0.001		*P* < 0.001		*P* < 0.001	
**Main effect**							
Aeration	No	70.6	7.5	5.96	1.29	1100	118
	Yes	28.5	3.8	8.97	1.81	1375	131
Temperature	RT	56.3	6.2	4.88	0.73	1022	106
	4°C	42.8	5.2	10.05	1.87	1453	143
**Two-way ANOVA (P)**	Aeration	*P* < 0.001		*P* < 0.01		*P* < 0.05	
	Temperature	*P* < 0.05		*P* < 0.001		*P* < 0.01	
	Aeration x Temperature	*P* > 0.05		*P* > 0.05		*P* > 0.05	


*Means in the same column not sharing same letters are significantly different at the level of P < 0.05*.

### Pollen Grain Morphology Following Ultrasonication Treatment

**FIGURE 4:**
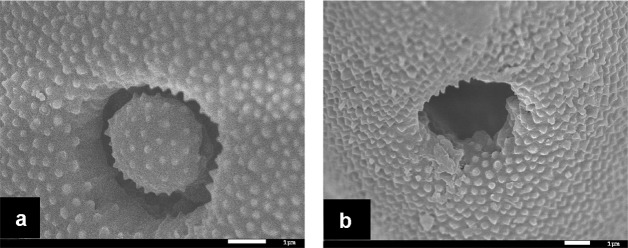
**Scanning electrical microscopic (SEM) micrographs of maize pollen grains. (a)** a pollen grain suspended in a 15% sucrose solution in the control group without ultrasonication, showing the presence of an operculum covering the pore; **(b)** a pollen grain suspended in a 15% sucrose solution at 4°C and treated with ultrasonication at the power of 150 W for 10 s with an interval of 10 s for six times, showing the absence of the operculum leading the wide opening of the pore.

Under normal conditions as shown in the control group that was not treated with ultrasonication in the current study, the germination pore of maize pollen grains was covered by an operculum as shown by scanning electrical microscopic image in **Figure 4a**. However, operculums of some pollen grains were removed following ultrasonication treatments. (**Figure 4b**). In the present study, of 225 pollen grains observed, 3 were found with removed operculums which accounted for 1.33%.

### Expression of *eGfp* in the Reproductive System

**FIGURE 5:**
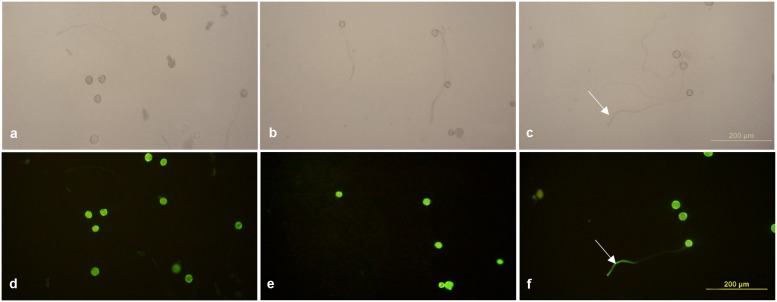
**Expression of *eGfp* in pollen tubes germinated from transformed pollen grains. (a,d)** Fresh pollen grains suspended in 15% sterilized sucrose solution; **(b,e)** germinated pollen grains incubated with recombinant plasmid containing *eGfp* gene without ultrasonication treatment applied; **(c,f)** germinated pollen grains incubated with recombinant plasmid containing *eGfp* with ultrasonication treatment applied. **(a**–**c)** Photographed under bright field with white light illumination; **(d–f)** photographed under blue light illumination. *EGfp* expression is visible in a pollen tube (white arrows shown in **c,f**) of the ultrasonication treated pollen grains, whereas no *eGfp* expression was observed in the pollen tubes of untransformed pollen grains, although pollen tubes are clearly visible under while light illumination. Pollen suspension from all treatment groups were incubated at 25°C.

**FIGURE 6:**
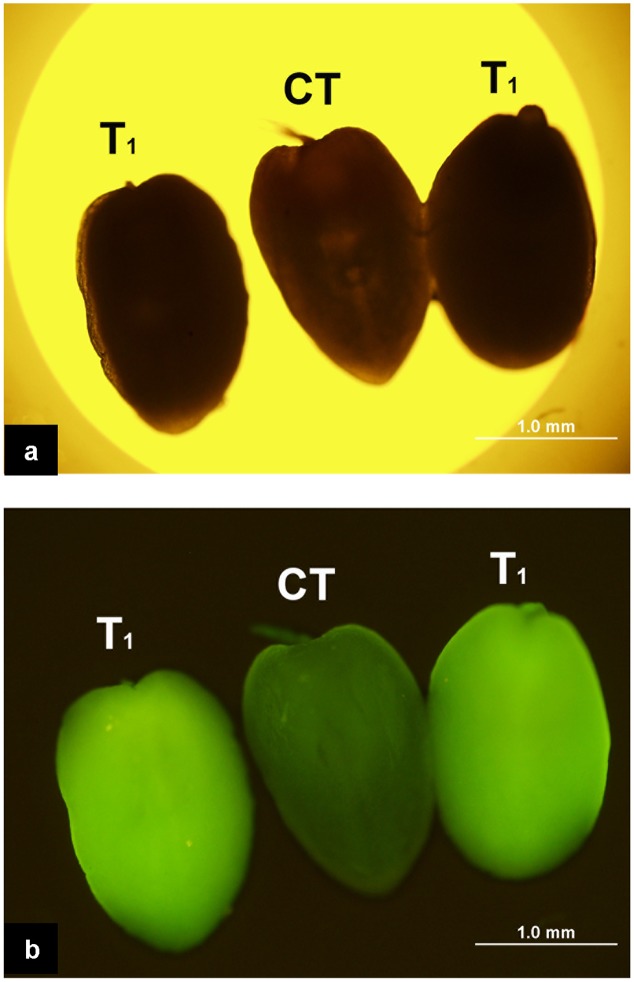
**Expression of *eGfp* in the embryos following the pollination of the plants with *eGfp*-transformed pollen grains. (a)** Embryos photographed under bright field with white light illumination. All three embryos are indistinguishable morphologically under such illumination; **(b)** Embryos photographed under blue light illumination. Two embryos on both sides produced from ears pollinated with pollen transformed with *eGfp* gene show the green fluorescence suggesting the expression of *eGfp*; the embryo in the middle (CT) produced from pollination with untransformed pollen does not show fluorescence indicating the absence of *eGfp* expression.

The enhanced *Gfp* gene (*eGfp*) cloned in the plasmid construct pLM01 and driven by the double CaMV 35S promoter (P35S promoter) was transformed to pollen grains following the ultrasonication treatments. The expression of *eGfp* was observed in a pollen tube germinated from the transformed pollen grain. No *eGfp* expression, however, was detected in the pollen tubes germinated from the pollen grains in the untransformed pollen, although pollen tubes were clearly visible (**Figure 5**). Following controlled pollination of the *eGfp* transformed pollen treated by ultrasonication, the expression of *eGfp* in the embryos of 15 days post pollination was observed, and the average transformed embryos accounted for 33.3% (**Table 2**) whereas the embryos from untransformed pollen did not show *eGfp* expression from all the plants (**Figure 6**).

**Table 2:** Summary of eGfp observed in embryos.

Replicate	No. of observed embryos	No. of transformed embryos	Transformation frequency (%)
1	15	6	40.0
2	8	2	25.0
3	10	3	30.0
**Average transformation frequency (%)**		33.3


### Detection of *Cry1Ac* or *Apr* Gene in the Leaves of T_1_ and Its Progeny Plants

**FIGURE 7:**
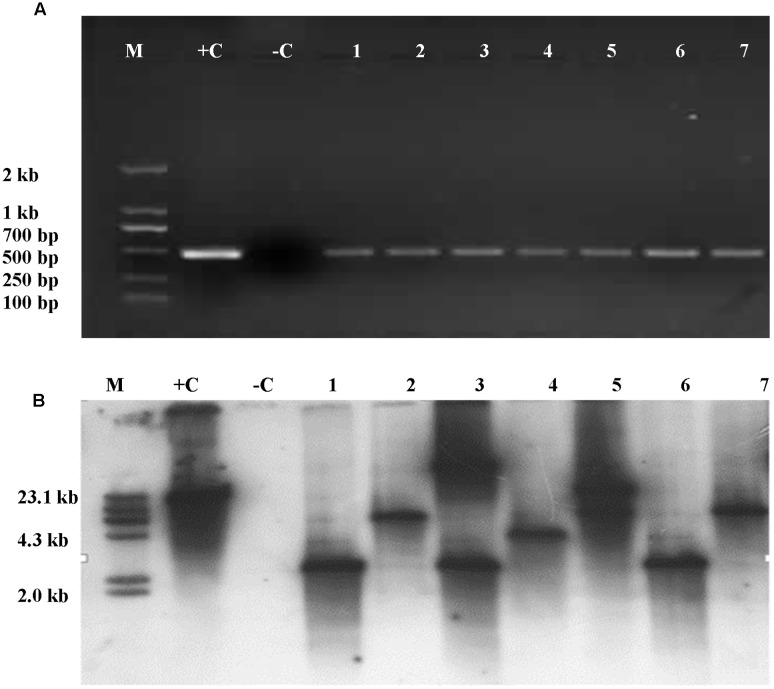
**Detection of *Cry1Ac* gene was analyzed in the leaves of T_1_ and its progeny plants through PCR amplification and Southern blot. (A)** PCR amplicons as part of *Cry1Ac* gene. M, DNA molecular standard with bands labeled in base pairs; +C, positive control with plasmid P3301UbiAc DNA as PCR template; –C, negative control with total genomic DNA of untransformed maize inbred line Zheng58 as PCR template; 1–7, transformed plant individuals. **(B)** Southern blot analysis of T_2_ individuals positive to PCR analysis. M, DNA molecular weight standard labeled with DIG; +C, positive control with plasmid P3301UbiAc DNA digested with *Hind* III; –C, negative control with total genomic DNA of untransformed maize inbred line Zheng58 as PCR template; 1–7, individuals (1, 3, and 6), 4, and (2, 5, and 7), respectively, are from three different lines of T_1_. Genomic DNA of the PCR-positive and control plants was digested with *Hind* III **(B)**.

Presence of *Cry1Ac* or *Apr* gene was analyzed in the leaves of T_1_ plants. It was shown that the *Cry1Ac* or *Apr* gene was detected in the leaves of T_1_ plants by PCR amplification. However, the primers were not able to amplify the gene in the untransformed maize inbred lines. The frequencies of transformed T_1_ plants were summarized in **Table 3**. Insertion of *Cry1Ac* gene in the maize genome was identified by Southern blot analysis of individuals produced from self-pollinated T_1_ plants with the probe specific to the gene, but no such hybridization was observed when the untransformed maize inbred line Zheng58 was used in the assay (**Figure 7**). Segregation in T_2_ seedlings analyzed by PCR also confirmed the stable transmission of foreign genes via pollen-mediated genetic transformation (**Table 4**).

**Table 3:** Summary of transformation frequencies of T_1_ plants.

Transformed plasmid	Receptor	Gene detected	No. of PCR positive plants / plants analyzed
pLM01	Zheng58	*Apr*	4/22
pLM01	Dika527	*Apr*	8/25
p3301UbiAc	Zheng58	*Cry1Ac*	24/74
**Average frequencies of transformed T_1_ plants (%)**			29.8


**Table 4:** Segregation of the *Apr* gene in T_2_ seedlings^a^.

	No. of seedlings				
Line	PCR positive	PCR negative	Ratio (positive/negative)	Expected	χ^2^
T2001	142	58	2.45:1	3: 1	1.00^b^
T2002	138	108	1.28:1	3: 1	45.88
T2004	136	56	2.43:1	3: 1	1.00^b^


^a^*T_2_ progeny was from the self-pollination of selected T_1_ plants*.

^b^*Indicated significance fit the expected segregation ratio (p < 0.05)*.

## Discussion

In the current study, we demonstrated that the foreign genes were transformed through pollen-mediated transformation method following ultrasonication treatment and expressed in the pollen tubes, embryos and progenies after transformation with the optimization of the pre-sonication treatment by aeration for 20 min at 4°C.

Male sexual reproduction system is an ideal pathway for genetic transformation. In animals, use of reproductive system in transgenic manipulation has been performed more than two decades ago (Wagner and Hoppe, 1989), and lately such gene transfer technique has been widely used in different animals including livestock (Nagano et al., 2001; Oatley et al., 2004). In plants, attempts to use pollen as a pathway for genetic transformation have been reported by Kumlehn et al. (2006) and Shim et al. (2009). However, an efficient way for the production of transgenic plants that can be widely applied in the breeding program is needed. The present study demonstrated the effectiveness of pollen-mediated transformation, elucidated the possible mechanisms underlying the transformation, and presented evidences of such transformation at molecular and histological levels. These suggest that the method has a potential role in plant improvement due to its merits: i.e., bypassing tissue culture procedure, simple to operate and flexible in its applications in different species and varieties.

Ultrasonication has been used in a few studies to treat tissues or protoplasts for easy access to the genome of recipients by foreign DNA molecules in plants (Zhang et al., 1991; Joersbo and Brunstedt, 1992; Wang et al., 2008; Zhang et al., 2015) and animals (Bao et al., 1997; Miller et al., 1999; Huber and Pfisterer, 2000; Hirabayashi et al., 2005). It has been proposed that ultrasonic waves produce high pressure and high temperature lead to the localized rupture of cell wall and thus enhance permeability of the plasma membrane (Liu et al., 2006; Wang et al., 2008). They also suggested transient cavitation produced by sonication may have enhanced the entry of foreign DNA into pollen. Here, we demonstrated with scanning electrical microscopy that the operculum covering the pollen germination pore was removed by ultrasonication and at least in some pollen grains, this made them become more competent to receive foreign DNA. We observed the expression of *eGfp* in the major part of the pollen tube. This at least indicates the transgenicity of the vegetative cell. We also found that the expression of *eGfp* in pollen tube appeared to restrict to the tip of pollen tube where vegetative nucleus resides along with the two sperm cells. We are unable to distinguish whether *eGfp* is expressed in the vegetative cell or the generative/sperm cells or both. Since the vegetative nucleus does not contribute to the zygote formation, we suggest that the transmission of foreign genes such as *eGfp* and *Cry1Ac* to T_1_ and T_2_ generations is likely through the generative/sperm cells given that these cells directly contribute to the formation of the zygote. Eventually, the transgene is passed onto the progeny by the kernel containing embryo developed from the zygote as evidenced by the *eGfp* expression in T_1_ embryos and *Cry1Ac* gene presence in the leaves of T_1_ plants.

These transformed pollen grains perform as vectors to introduce exotic genetic materials into the genome of recipient plants following pollination and fertilization of theirs eggs. To the best of our knowledge, this is the first direct evidence to show how sonication acts on its biological target to make it permeable to allow the uptake of DNA molecules from the surrounding solution. Furthermore, this also indicates that the presence of pollen pore might be the reason to enable pollen to be used as a vector to mediate genetic transformation despite its presence of protective exine wall.

To understand the underlying mechanisms of this transformation system more thoroughly, subsequent studies are warranted to investigate the process how the foreign genes are transferred to generative/sperm cells employing, for instance, immuno-gold labeling at scanning electron microscopy (SEM) or TEM level, what is the frequency of such transformation relative to that to vegetative cell and the possible copy numbers of genes transferred to the generative/sperm cells through immuno-histochemical and genetic approaches.

In the present study, optimal pre-treatment of pollen grains by aeration of 20 min at 4°C was applied as the aeration and low temperature led to better pollen viability and kernel set. We also observed that fresh pollen could be better kept with the presence of drierite. Such improved pollen grain viability may have helped to some extent the introduction of foreign genes into the recipients due to higher successful pollination and thus improved kernel set during the experiment. Although we used field grown plants as pollen donors in the study, we observed that pollen viability can largely be maintained as long as pollen is collected in clear days according to our previous experiences over years. Therefore, the method is fairly robust and can be readily used by the breeders who do not have expensive greenhouse facilities. Nevertheless, the efficiency of the method can be improved under controlled conditions.

The pollen-mediated transformation system developed by our group has been proved effective in delivering foreign genes to directly generate transgenic kernels without using tissue culture procedures. The optimization of treatment conditions to pollen grains prior to sonication makes it more efficient. This system has many advantages over traditional tissue culture-based transformation system, i.e., its simplicity, fast turnaround, low costs, so that breeders can utilize such transformation system in their breeding program without excessive efforts of training and need of expensive equipment. We believe this pollen-mediated transformation system is a promising tool and will play an important role in plant breeding area.

## Author Contributions

LY performed SEM and fluorescence microscopy observations with *eGfp* expression and pollen morphological analysis, and jointly drafted the manuscript; GC optimized sonication treatments of pollen grains; YW performed PCR and Southern analysis; JD, HZ, YH, and HZ participated pollen transformation, pollination and data collection; CW managed field treatment, observation and data collection; S-BW participated experiment design, performed data analysis, and jointly drafted manuscript; YS conceived of, designed and coordinated the study, analyzed the data, and critically revised the manuscript. All authors have read and approved the final manuscript.

## Conflict of Interest Statement

The authors declare that the research was conducted in the absence of any commercial or financial relationships that could be construed as a potential conflict of interest.
